# Malignant distal femur tumors management in children, low cost innovations with affordable care in a tertiary care hospital, a cross sectional study

**DOI:** 10.1016/j.amsu.2019.07.012

**Published:** 2019-07-10

**Authors:** Masood Umer, Javeria Saeed, Obada Husseinali

**Affiliations:** Department of Surgery, Aga Khan University Hospital, Stadium Road, P. O. Box 3500, Karachi, 74800, Pakistan

**Keywords:** Distal femur, Malignant tumors, Revision surgery, Wound infection, Broken plates

## Abstract

**Introduction:**

Malignant musculoskeletal tumors in children are mostly comprised of Osteosarcoma and Ewing's sarcoma and distal femur is the most common site for primary bone tumors. This study examined the surgical management outcomes of pediatric patients (<18 years) presented in our setup with malignant distal femur tumors.

**Methodology:**

We retrospectively reviewed the medical records of patients diagnosed with malignant sarcomas of distal femur and younger than 18 years of age who underwent limb salvage surgery during June 2009–June2017.

**Results:**

There were 31 pediatric patients who had distal femur malignant tumors and out of them 20 patients were selected who underwent limb salvage as a primary surgery. The mean age (range) of patients was 11.90 (6–17) years. Seventeen had osteosarcoma and three had Ewing's sarcoma. In surgery all patients were offered reconstruction. Post-operative complications were observed in (50%) patients while other 50% patients had no complications. Wound infection, flap necrosis, broken plates with non-union and recurrence of disease were the reported complications. Revision surgery was performed in 10 patients out of 20 patients.

**Conclusion:**

Salvage was the primary option in our surgery and revision surgery was performed in the cases due to complications. Small sample size was the limitation of the study.

## Highlights

• Distal femur tumors in pediatric population can be managed with low cost.
• The innovations discussed in study include fibular grafting, autoclaved tumor bone and parental fibula.
• In resource retrained countries, multiple reconstruction options are available with  good outcome for the children.

## 1 Introduction

Malignant bone tumors are disease of the young bone. Pediatric patients suffer the maximum burden of primary bone tumors [1]. It's a great challenge for the surgeon, oncologist and physiotherapist to deal in management of this age group. Furthermore, the psychosocial impact on the child and the parents can't be ignored. Fortunately, in the last 3 decades there was dramatic increase in the 5 year survival rates of these tumors, from 10% to over 70%, thanks to the combination of new chemotherapeutic agents, advanced reconstruction techniques and the affordable, effective and durable alternates at resource constrained country like ours [2]. This survival is long enough to justify the complex procedures in limb sparing surgery. At this era, limb-salvage procedures are the gold standard for most of the cases [3]. Amputation is recommended in very selective patients and cases.

Malignant musculoskeletal tumors in children are mostly comprised of Osteosarcoma and Ewing's sarcoma. Among all malignancies excluding leukemia and lymphoma, Osteosarcoma comprises of 3–5% cases of malignant tumors in children [4]. The distal femur is the most common site involved [5]. Not only is this an anatomically critical site involving a significant joint, but also, being in lower limb, hinders the weight bearing and functional status of the child. Managing such tumors require a highly professional team compromising of an orthopedic tumor surgeon, oncologist, pediatrician, radiotherapist, physiotherapist and nurses [6]. Growing skeleton of the child adds to the challenge. You won't be able to use fixed, rigid prosthesis as used in adults. Dynamic growing prostheses are too expensive for our patients who pay their health care services out of their pockets [7]. At our side of the world, multiple cost-effective alternates have been in use [8]. These include parental fibula, vascularized autografts and autoclaved tumor bone. Vascularized free fibula grafting was introduced in 1970 for reconstruction in bone tumors and it was shown to have benefits to patients receiving radio/chemotherapy. This type of free fibula grafting also remains viable in infections. Although they possess the ability to unite and remodel with the host bone and increase in strength, free fibulas initially lack the structural support of large cortical allografts [9].The present study is conducted to share our experience of management of malignant distal femur tumor in children and show the low cost innovations with affordable care.

## 2 Methodology

A retrospective cross sectional study was planned and the medical record files of all the patients diagnosed with malignant sarcomas of distal femur and younger than 18 years of age, who underwent limb salvage surgery in Aga University hospital during June 2009–June2017 were reviewed. It was a single centered study. Adult Patients (>=18 years) that were diagnosed with any benign bone tumors and other than distal femur site with malignant tumor diagnosis were excluded from the study. Study protocol was submitted in ERC and was granted exemption from hospitals Ethics review committee as it was a retrospective audit.

Data was extracted from patient's medical record file and entered on statistical software SPSS 21. A structured proforma was used for data collection. The data was collected for the following variables; demographics including: age, gender. Moreover, information was also taken on tumor site, surgery details, reconstruction, and histopathology, complications, neo-adjuvant and adjuvant therapy. Since it was retrospective audit so the patients last follow up was recorded only and no further follow up was done. All the descriptive analysis of data was done and results were reported as frequencies and mean. This study has been registered in research registry, number is researchregistry4695 and it is being reported in line with STROCSS research reporting guidelines [10].

## 3 Results

There were 31 pediatric patients who had distal femur malignant tumors and out of them 20 patients were selected who underwent limb salvage as a primary surgery. The mean age (range) of patients was 11.90 years (Table 1). There were 11 females (55.0%) and 9 males (45%). Seventeen patients (85.5%), had osteosarcoma and three (15%) had Ewing's sarcoma. Patients were treated with surgery, neo-adjuvant and adjuvant chemotherapy. All patients had neo-adjuvant and adjuvant chemo therapy which was given according to EURAMOS and COG protocol, while only 1 patient underwent adjuvant radiotherapy. In surgery all patients were offered reconstruction.

**Table 1:** Age descriptives of patients (Years).

n	Minimum	Maximum	Mean	Std. Deviation
20	6	17	11.90	3.227

The reconstruction method used were: vascular fibula with platting (single, double fibula), mega prosthesis insertion, patients, autoclave bone and allograft from parental fibula (Table 2). 12 patients had right distal femur disease (60.0%) and 8(40.0%) had left side affected.

**Table 2:** Methods of reconstruction.

Type of reconstruction	Frequency	%
Vascular fibula with platting	13	65.0
Mega prosthesis	2	10.0
Autoclave bone	4	20.0
Allograft	1	5.0
Total	20	100.0

**Fig. 1:**
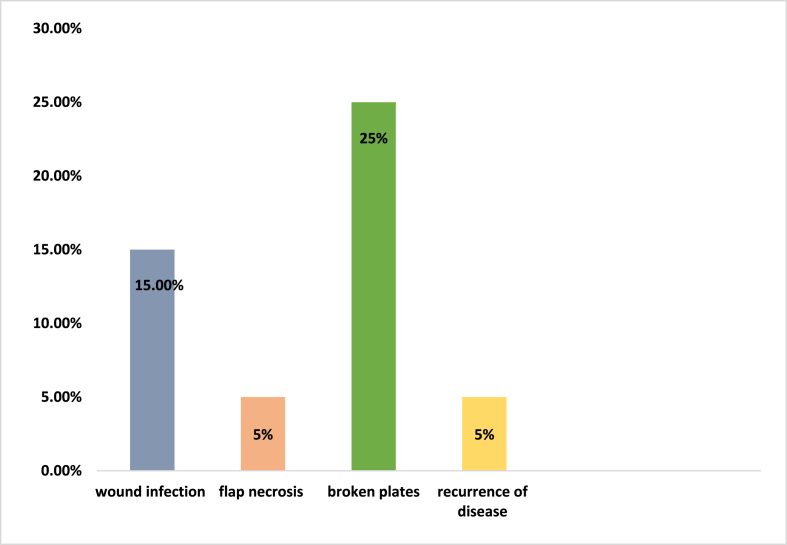
Types of post-operative complications.

Post-operative complications were observed in 10 (50%) patients while other 10 patients had no complications. Wound infection occurred in 3 patients, 1 patients had flap necrosis, and 5 had broken plates with non-union, while 1 had recurrence of disease (Fig. 1). Revision surgery was performed in 10 patients out of 20 patients. Amputation as a revision surgery option as a result of complications was done in 4 (20%) patients while 16 (80%) had other revision surgeries comprising of removal of broken implant, bone grafting with BMP (bone morphogenic protein) and wound debridement.

Post-operative complications were observed in 10 (50%) patients while other 10 patients had no complications. Wound infection occurred in 3 patients, 1 patients had flap necrosis, and 5 had broken plates with non-union, while 1 had recurrence of disease (Fig. 1). Revision surgery was performed in 10 patients out of 20 patients. Amputation as a revision surgery option as a result of complications was done in 4 (20%) patients while 16 (80%) had other revision surgeries comprising of removal of broken implant, bone grafting with BMP (bone morphogenic protein) and wound debridement.

## 4 Discussion

Bone and soft tissue sarcomas are rare mesenchymal malignancies that arise in 2–4 per 100,000 head of population [11]. Most common being Osteosarcoma and Ewing sarcoma. Affecting the distal femur most commonly. Worldwide there is a dramatic increase in 5-year survival rate in the last 3 decades after introducing newer chemotherapeutic agents, advances in imaging modalities and advanced training and surgical techniques in orthopedic tumor cases [11]. Even patients with advanced stage and those with metastases at time of diagnosis have better outcomes due to these chemotherapeutic agents and surgical excision of the primary tumor along with the metastases [12].

Surgery to resect the tumor followed by reconstructions to preserve function, mobility, and esthetics (limb-sparing surgery) has now replaced amputation as the primary form of surgical intervention [[13], [14], [15]]. All of our patients in current study offered reconstruction after wide margin excision. All had neo-adjuvant chemotherapy based on EURAMOS and COG protocols, while only 1 patient had adjuvant radiotherapy. Only 2 patients had the opportunity to have mega prosthesis. The maximum number of patients 13 had biologic reconstruction using vascularized fibular graft fixed with locking plate. Innovative techniques, based on the resource-constrained circumstances we have, were utilized in the remaining patients. Like allograft from parental fibula and autografts from autoclaved bone.

Complications such as periprosthetic fracture, breakage of plate, prominent implant, non-union at host-graft junction and bone and joint stiffness have all been reported in literature. In our series, we have encountered complications in 10 patients. Wound infection, flap necrosis, broken plates with non-union and recurrence of disease were also found in our patients. Revision surgery was performed in half of the patients. Amputation as a revision surgery option as a result of complications was done in 4 patients while 16 had other revision surgeries comprising of removal of broken implant, bone grafting with BMP (bone morphogenic protein), and wound debridement and distraction osteogenesis by ilizarov apparatus.

Not only we have scarcity of relevant published literature and health care rules and regulations which are near to minimum, but also the inadequate awareness and limited diagnostic and therapeutic specialized facilities make it real challenging to manage such tumors at our setup.

Definite diagnosis is mandatory before any attempt at surgical intervention. In reality, this sometimes is difficult and patients with malignant bone disease can be misdiagnosed as having benign lesions. This is a rare disease you won't encounter much in your practice as a general orthopedic surgeon so it's always helpful to follow international guidelines like the National Comprehensive Cancer Network (NCCN). They recommend referring all patients younger than 40 years of age who present to you with bone pain, and a suspicious lesion on x-ray, to an orthopedic oncologist for biopsy and further management.

## 5 Conclusion

The increase in awareness of the patients and the surgeons towards this highly sophisticated subspecialty will make early referral to specialized centers easier task, hence the better long term outcome for these children. The limitations of this study was a retrospective data collection which didn't allow us to find the present survival status of patients. We emphasize that the experts who received advanced training in Orthopedics tumor surgery should deal such cases. Orthopedics societies should guide surgeons to refer such cases to the concerned to save the lives and limbs of our patients. Multiple options of reconstructions are available. In resource-constrained settings, there are affordable, durable and effective options of reconstructions as well. The multidisciplinary approach and team work yield the best results to spare the limb of these children and grant them a better quality life.

## 6 Declaration of inerest

None.

## 7 Research funding

This research did not receive any specific grant from funding agencies in the public, commercial, or not-for-profit sectors.

## Ethical approval

The study was granted exemption from hospital ERC, the Number is 4760-Sur-ERC-17.

## Provenance and peer review

Not commissioned, internally reviewed.

## Sources of funding

None.

## Author contribution

Masood Umer: proposed the idea of study and gave a final review to manuscript.

Javeria Saeed: data collection, analysis and manuscript writing.

Obada Husseinali: manuscript writing.

## Conflicts of interest

There is no conflict of interest.

## Research registry number

Researchregistry4695.

## Guarantor

Dr Masood Umer.
